# Enhancing enzymatic digestibility of waste wheat straw by presoaking to reduce the ash-influencing effect on autohydrolysis

**DOI:** 10.1186/s13068-019-1568-7

**Published:** 2019-09-17

**Authors:** Wei Tang, Xinxing Wu, Chen Huang, Caoxing Huang, Chenhuan Lai, Qiang Yong

**Affiliations:** 1 Key Laboratory of Forestry Genetics & Biotechnology (Nanjing Forestry University), Ministry of Education, Nanjing, 210037 People’s Republic of China; 2 Jiangsu Co-Innovation Center of Efficient Processing and Utilization of Forest Resources, College of Chemical Engineering, Nanjing Forestry University, Nanjing, 210037 People’s Republic of China; 3 Jiangsu Province Key Laboratory of Green Biomass-based Fuels and Chemicals, Nanjing, 210037 People’s Republic of China

**Keywords:** Presoaking, Cations, Waste wheat straw, Autohydrolysis, Enzymatic hydrolysis

## Abstract

**Background:**

The acid buffering capacity of high free ash in waste wheat straw (WWS) has been revealed to be a significant hindrance on the efficiency of autohydrolysis pretreatment. Previous researches have mainly relied on washing to eliminate the influence of ash, and the underlying mechanism of the ash influencing was not extensively investigated. Presently, studies have found that cations can destroy the acid buffering capacity of ash through cation exchange. Herein, different cations were applied to presoak WWS with the aim to overcome the negative effects of ash on autohydrolysis efficiency, further improving its enzymatic digestibility.

**Results:**

Results showed that cations can be adsorbed on the surface of the material by electrostatic adsorption to change the acid buffering capacity of WWS. The acid buffering capacity of 120 mM Fe^2+^ presoaked WWS is reduced from 226.3 mmol/pH-kg of original WWS to 79.3 mmol/pH-kg. This reduced the autohydrolysis pretreatment medium pH from 5.7 to 3.8 and promoted the removal of xylan from 61.7 to 83.7%. In addition, the enzymatic digestibility of WWS was enhanced from 49.7 to 86.3% by presoaking with 120 mM Fe^2+^ solution. The relationship between enzymatic accessibility and hydrophobicity with enzymatic digestibility of the autohydrolyzed WWS was analyzed.

**Conclusions:**

The results showed that the acid buffering capacity of the high free ash was detrimental for the autohydrolysis efficiency of WWS. After WWS was presoaked with different cations, the acid buffering capacity of ash was weakened by cation exchange and electrostatic adsorption, which improved the autohydrolysis efficiency. The results expound that the enzymatic digestibility of WWS can be enhanced through presoaking to reduce the ash-influencing effect on autohydrolysis.

## Background

To mitigate greenhouse gas emissions and defuse energy crisis, development of sustainable and economic biomass resources for biofuel production has become a major focus and essential for meeting the world’s energy demand [1–3]. Implementation of biorefinery processes that utilize lignocellulose from agriculture and forestry as feedstocks is paramount towards achieving the goal [4]. Challenging this implementation is the complexity of lignocellulosic cell walls, which, existing as a complex matrix of cross-linked biopolymers (cellulose, hemicellulose, and lignin), is difficult to disrupt [5, 6]. This organized and compact structure exists around the cellulose, which can only be bio-converted into its constituent glucose if sufficient structural disruption around cellulose’s surrounding biopolymers takes place [7]. Disrupting the complex structure of lignocellulosic materials by pretreatment is a necessary step for the biorefinery process. Pretreatment can improve the enzymatic hydrolysis of lignocellulose by reducing the recalcitrance for enzyme, such as hydrolyzing the hemicellulose, relocating the lignin, and changing the cellulose properties [8, 9].

Many pretreatment methods for lignocellulose have been developed, such as steam explosion, ammonia, alkali, and dilute acid pretreatment. However, these methods are difficult to be commercialized due to high energy requirements, high toxicity, and extreme pretreatment conditions [8, 10]. A green and promising pretreatment technology, autohydrolysis, exists as a more benign version of dilute acid pretreatment that does not employ addition of reagents beyond water [11]. Autohydrolysis is thus much less corrosive to process equipment, and its process intensity lowers the extent of enzyme and fermentative inhibitors [12]. It has been reported that autohydrolysis was also able to remove hemicellulose thus improving cellulose accessibility and digestibility by enzymes [13, 14]. The active ingredient of autohydrolysis comes in the form of hydronium ions produced when very labile acetyl groups of hemicellulose are cleaved during the pretreatment process. These ions lower the pH of the autohydrolysis medium to pH ~ 3–4, depending on feedstock’s degree of acetyl substitution [15]. Hydronium ions effectively promote hemicellulose depolymerization and solubilization in the form of its constituent monosaccharides and oligosaccharides [16].

Although the hemicellulose can be gently removed during autohydrolysis, it was found that the presence of inorganic ash in lignocellulose exerts a buffering effect on the autohydrolysis efficiency [17]. This effect was first noticed when studying autohydrolysis of waste wheat straw (WWS), which consisted of wheat ears, leaves, straw scraps and approximately ~ 30% free ash by weight [18]. The free ash engages with the hydrolysate as an acid buffer during pretreatment, causing ineffective autohydrolysis [19]. Eliminating the effects of free ash will be a requirement before agricultural residues like WWS can be utilized in biorefinery processes, mostly because these low-cost feedstocks demand biorefinery processing that is similarly low in cost (i.e., reagent-free autohydrolysis) [20]. The lignocellulosic ash presented in WWS mostly originated from the soil in which it resided prior to collection [18]. It can be roughly divided into organic and inorganic solids. The inorganic phase is comprised of insoluble inorganic minerals and surface sites occupied by cations like H^+^, K^+^, Na^+^, Ca^2+^ and Mg^2+^ [21]. It is the exchange of these alkaline ions that is believed to the cause of acid buffering during autohydrolysis [19]. In fact, it has been shown that the cations in the ash of corn stover showed the buffering potential during autohydrolysis pretreatment, which can be eliminated by room temperature sulfuric acid washing prior to pretreatment for ensuing effective autohydrolysis [22]. In addition, the cations adsorbed on the surface of ash in lignocellulose can be exchanged with the invading metal cations based upon the strength of cation exchange capacity to reduce its acid buffering capacity [23].

It has been shown that employing Fe^3+^ or Al^3+^ in autohydrolysis process can overcome the ash self-buffering effects. This approach enhanced enzymatic digestibility of autohydrolyzed WWS from 49.7 to 66.6% [19]. The improvement was attributed to the cations in the system that could exchange with the buffering cations on the surface of free ash, resulting in dampening buffering capacity of ash [19]. However, the unexchanged (free) metal salts added during the pretreatment problematically catalyzed formation of fermentation inhibitors from monosaccharides [24, 25]. An alternative method called presoaking can also counter the buffering cations of free ash without inducing unwanted catalysis of carbohydrates to inhibitory species and avoiding the corrosive effects noticed when adding the acidic reagents [26]. Therefore, the aim of this paper was to improve autohydrolysis and subsequent enzymatic digestion of WWS by implementing a benign presoaking stage intended to neutralize the buffering effects of the free ash present within it.

In this work, the WWS was presoaked with different cations (H^+^, K^+^, Na^+^, Ca^2+^, Mg^2+^, Zn^2+^, Fe^2+^) solutions to reduce the buffering effect of ash on autohydrolysis. The presoak solution was prepared with or without different types of cations. The surface charge, acid buffering capacity, and cation exchange capacity of the presoaked WWS were investigated to show the degree of disintegration and degree of reduction of the buffer system. In addition, the accessibility and hydrophobicity of autohydrolysis WWS were determined to estimate the effects of presoaking with cations on the structural properties of WWS.

## Materials and methods

### Materials

WWS was roughly screened from wheat straw used for pulping, which was kindly provided by Quanlin straw pulp mill in Shandong province, China. The dry matter content of WWS was 86 wt% determined by an Infrared Moisture Balance (FD-720, KETT, Japan). Cellulase (Cellic^®^ CTec2) was purchased from Novozymes North America (Franklinton, NC, USA) with 150 mg protein/mL. K_2_SO_4_, Na_2_SO_4_, CaSO_4_·2H_2_O, MgSO_4_, ZnSO_4_·7H_2_O, and FeSO_4_·7H_2_O were each purchased from Nanjing Chemical Reagent Co., Ltd (Jiangsu province, China). All reagents used in this work were of analytical grade.

### Presoaking WWS with different cations

**Fig. 1:**
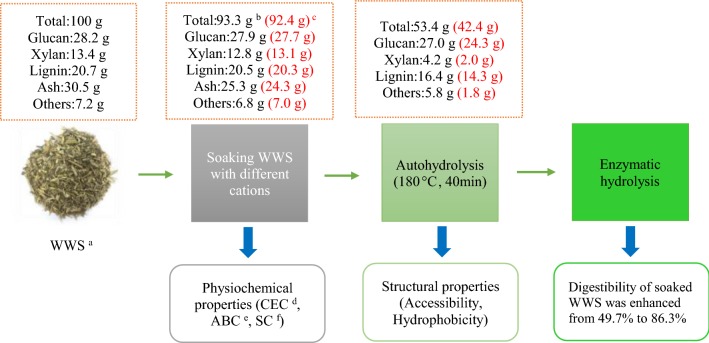
The scheme of autohydrolysis and enzymatic hydrolysis of WWS with presoaking. ^a^ waste wheat straw; ^b^ WWS presoaked with water; ^c^ WWS presoaked with 120 mM Fe^2+^ and the results were showed in red brackets; ^d^ cation exchange capacity; ^e^ acid buffering capacity; ^f^ surface charge

150 g dry weight of WWS was soaked with 4.5 L of 30 mM of different cations solutions (H^+^, K^+^, Na^+^, Ca^2+^, Mg^2+^, Zn^2+^) and different concentrations of Fe^2+^ (10 mM to 120 mM) at room temperature for 24 h. Soaking with water alone was also performed as a control group. After soaking, the slurry was filtered by passing it through a cloth bag while retaining solid WWS. Finally, the physiochemical properties of the presoaked WWS were separately determined and then autohydrolysis was performed. The specific experimental steps are shown in Fig. 1.

### Physicochemical properties of presoaked WWS

#### Surface charge of presoaked WWS

Surface charge (SC) of presoaked WWS was assessed by measuring zeta potential. Presoaked WWS was crushed with a lapping machine (Traditional Chinese Medicine Grinder, RS-FS1401) and screened over a 200-mesh sieve and next suspended in 50 mL of deionized water (2% w/v) for zeta potential determination. The suspensions were fragmented by a homogenizer at 5000 rpm for 5 min to remove insoluble solids. The supernatant was then separated and scanned for zeta potential using an SZP meter (Mütek SZP 06) [27]. Three replicates were tested for each sample and the results are presented as an average.

#### Cation exchange capacity of presoaked WWS

The cation exchange capacity (CEC) was determined by the forced exchange method employing BaCl_2_–H_2_SO_4_ [28]. Specifically, 1 g dry weight of each presoaked sample’s ash was suspended in 30 mL of BaCl_2_ solution (0.1 M). Each suspension was then centrifuged at 5000 rpm for 10 min, and the supernatant was discarded. This operation was repeated three times to thoroughly exchange cations on the ash surface. 50 mL of deionized water was finally used to remove remaining Cl^−^. This water washing step was also repeated three times. Next, each solid fraction was suspended in 25 mL H_2_SO_4_ solution (0.1 M) and shaken for 15 min. After that, the mixture was filtered with triple layer filter paper (11 cm slow speed, Fushun, China), and the filtrate was then titrated with 0.1 M NaOH solution to calculate H_2_SO_4_ consumption. The value of CEC was calculated from the amount of H_2_SO_4_ adsorbed by per gram of ash in presoaked WWS.

#### Acid buffering capacity of presoaked WWS

Acid buffering capacity (ABC) was determined using the pH buffering capacity method reported in literature [29]. Specifically, 10 g dry samples were placed into a polytetrafluoroethylene crucible (50 mL PTFE, JINGYIN, China) and burned in a muffle furnace at 575 °C for 12 h. After incineration, 1 g dry weight of the burned ash obtained was accurately massed and then suspended in 50 mL deionized water. The suspension was titrated with 0.1 M H_2_SO_4_ solution at a flow rate of 0.05 mL/min using a multi-water quality meter (MM-60R, TOADKK, Tokyo, Japan), and the terminal pH value was set at 3.0. The ABC of presoaked WWS was calculated from the consumption of H_2_SO_4_ during the pH reduction from 6 to 3 [18].

#### Autohydrolysis of presoaked and raw WWS

Prior to autohydrolysis, the pH of presoaked WWS was adjusted to 6.5 ± 0.2 as same as the pH of the raw WWS using concentrated NaOH solution. The presoaked WWS was next subjected to autohydrolysis in sealed vessels heated by an electrically heated oil bath (HH-SJ6CD, YOULIAN, China) and tumbling agitation. Various parameters of the pretreatment were controlled with a modular controller. Specifically, 50 g of presoaked WWS was added into 1.25-L container to achieve a final solid-to-liquid ratio of 1:10 (w/v). For pretreatment, the reactor temperature was raised from 60 to 180 °C at a heating rate of 2 °C/min over autohydrolysis time ranging from 0 to 40 min [30]. At the conclusion of autohydrolysis, the container was immediately cooled using water. Autohydrolyzed residues were separated from the liquid hydrolysate with the cloth bags used in presoaking, and then the collected residues were washed to neutral using deionized water and finally stored in sealed plastic bags kept at 4 °C. The composition of the separated liquid prehydrolysate was analyzed to evaluate the pretreatment efficiency.

### Chemical composition analysis of autohydrolyzed substrates

The chemical composition of WWS was analyzed using the National Renewable Energy Laboratory (NREL) standard method [31]. All prehydrolysate and enzyme hydrolysate samples were diluted with deionized water and filtered through a 0.22 μm filter. The xylooligosaccharides (XOS) of prehydrolysate were hydrolyzed into xylose by 4 wt% H_2_SO_4_ at 121 °C for 1 h, and its concentration was calculated by the change of the xylose concentration before and after the acid hydrolysis. The monosaccharides and inhibitors present in each solution were analyzed by a HPLC system (Agilent 1260 series, Agilent Technologies, USA) with a Coregel-87H column and the temperature was held at 55 °C. 0.05 M H_2_SO_4_ was used as mobile phase at a flow rate of 0.6 mL/min and the quantification was analyzed based on a refractive index detector. The ash content of WWS was also determined using the National Renewable Energy Laboratory (NREL) standard method [31]. 10 g dry weight samples were placed into a polytetrafluoroethylene crucible and burned in a muffle furnace at 575 °C for 4 h. The burning residue was considered to be the ash of the material. Each experiment was run in duplicate.

Recovery yields of carbohydrates and degree of delignification were calculated according to the following equations:

$${\text{Glucan}}\;{\text{recovery}}\;{\text{yield}} \;\left( \% \right) = \frac{{{\text{glucan}}\;{\text{in}}\;{\text{autohydrolyzed}}\;{\text{WWS}}\;{\text{residue }}\left( {\text{g}} \right)}}{{{\text{glucan}}\;{\text{in}}\;{\text{raw}}\;{\text{WWS}} \left( {\text{g}} \right)}} \times 100\%$$

$${\text{Xylan}}\;{\text{or}}\;{\text{lignin}}\;{\text{removal}} \;\left( \% \right) = \left( {1 - \frac{{{\text{xylan}}\;{\text{or}}\;{\text{lignin}}\; {\text{in}}\;{\text{autohydrolyzed}}\;{\text{WWS}}\;{\text{residue}} \left( {\text{g}} \right)}}{{{\text{xylan}}\;{\text{or}}\; {\text{lignin}}\;{\text{in}}\;{\text{raw}}\;{\text{WWS}} \left( {\text{g}} \right)}}} \right) \times 100\% .$$

### Structural property of autohydrolyzed WWS

#### Cellulose accessibility

The Direct Red 28 dye adsorption assay for determining the accessibility of cellulose to cellulases was performed as described in literature [32]. 1% of autohydrolyzed WWS was suspended in glass bottles containing 20 mL of distilled water. Next, a series of solutions containing increasing amounts of Direct Red 28 dye was added (0, 0.05, 0.1, 0.5, 1.0, 2.0, 3.0, 4.0 g/L). Each bottle was incubated for 24 h in a rotary shaker (Vortex-2, Shanghai, China) at 60 °C and 150 rpm. After that, the samples were centrifuged at 3000 rpm for 5 min. The supernatant absorbance of the sample and a standard solution was determined by ultraviolet–visible spectrophotometry at 498 nm. The amount of dye adsorption was calculated according to the absorbance reading, and a Langmuir adsorption isotherm was constructed to describe the behavior of direct adsorption on the tested substrates. The formula is as follows:

$$A = \frac{{A_{\hbox{max} } KC}}{1 + KC}$$

where *A* is the corresponding adsorbed dye (mg/g), *C* is the free dye in supernatant (g/L), *A*_max_ is the maximum adsorption capacity, and *K* is the Langmuir constant.

#### Hydrophobicity

The hydrophobicity of the autohydrolyzed residues was estimated using the Rose Bengal reagent dyeing method [33]. Specifically, different concentrations of each sample (0.04, 0.08, 0.12, 0.16, 0.2 g/L) were added into 50-mL screw-capped glass bottles followed by addition of 20 mL of Rose Bengal solution (40 mg/L) in citrate buffer (50 mM, pH 4.8). The bottles were incubated at a constant temperature of 50 °C with 150 rpm agitation in a rotary shaker. After 2 h, the suspensions were centrifuged at 5000 rpm for 5 min. Residual dye in the supernatant was detected at 543 nm using a UV–visible spectrophotometer. The amount of the dye absorbed by substrate was obtained by subtracting the amount of residual dye detected in the supernatant from the initial amount of dye added. The ratio of the amount of adsorbed dye to the amount of free dye remaining was also linearly fitted to the concentration of the added material to obtain a slope that describes each substrate’s hydrophobicity.

### Enzymatic hydrolysis of autohydrolyzed WWS

The autohydrolyzed WWS was enzymatically hydrolyzed using CTec2 at a substrate concentration of 5% (w/v) in 150 mL bottles. The pH of the hydrolysis reaction slurry was maintained at 4.8 using a 0.05 M citrate buffer, and the amount of cellulase added was 15 mg protein/g glucan. The reaction slurry was then shaken at 150 rpm in a constant temperature oscillator at 50 °C with liquid sampling at 2, 4, 6, 12, 24, 48, and 72 h. 0.2 mL of samples collected at different time intervals was centrifuged at 10,000 rpm for 5 min to obtain the supernatant which was analyzed by HPLC. Hydrolysis yields were calculated based on the released glucose as a percentage of the theoretical glucose available in the substrates. The corresponding formula is as follows:

$${\text{Glucan}}\;{\text{conversion}} \left( \% \right) = \frac{{{\text{glucose}}\;{\text{in}}\;{\text{enzymatic}}\;{\text{hydrolysate}} \left( {\text{g}} \right)}}{{1.10 \times {\text{glucan}}\;{\text{in}}\;{\text{raw}}\;{\text{substrate}} \left( {\text{g}} \right)}} \times 100\% .$$

## Results and discussion

### Physicochemical properties of presoaked WWS

The cation exchange capacity and acid buffering capacity are two crucial properties of soil ash [34]. The ABC of ash is defined as its resistance to changes in pH when acid was added (mmol/pH-kg), which contributed to the low efficiency of WWS autohydrolysis [18]. It has been reported that the CEC has a positive correlation to the given sample’s ABC [29]. Cation exchange is a reversible reaction in ash solution, dependent upon negative charges of ash components arising from charged or pH-dependent sites on the mineral and organic colloidal particles surfaces [34]. Invading cations can be absorbed by the ash dependent upon electrostatic force to exchange the dissociable cations that existed on the surface of ash [35]. Thus, the SC, CEC, and ABC of the presoaked WWS were determined to evaluate the modification of WWS by presoaking.

As shown in Table 1, the SC of the presoaked WWS weakened from the original − 25.9 mV to varying extents due to the presoaking using different types of cations. The effect of Fe^2+^ was the most prominent on reducing the SC to − 11.1 mV. When the Fe^2+^ concentration increased from 10 to 120 mM, the SC of WWS decreased from − 24.4 to − 4.66 mV. As expected, the negative surface potential of ash was bombarded by the introduction of positive valence cations according to electrostatic force, causing the SC to be gradually neutralized as the amount of cations added increased [36]. In Table 1, it also can be seen that the CEC value of the raw WWS could be reduced from 140.2 to 80.8–133.7 cmol/kg when it was presoaked with different cations. The Fe^2+^ applied in this work stood out amongst all cations, which reduced the CEC into 80.8 cmol/kg. The reason for these results maybe that the external cations adsorbed by electrostatic force changed the distribution of cations in the ash of WWS during presoaking which can result in a greater extent of reduction the CEC of ash in WWS. In addition, Table 1 showed that the ABC of raw WWS was 226.3 mmol/pH-kg, and the value decreased after soaking with 30 mM H^+^, K^+^, Na^+^, Ca^2+^, Mg^2+^, Zn^2+^ and Fe^2+^. When the concentration of Fe^2+^ increased from 10 to 120 mM, the ABC of WWS significantly decreased from 194.7 mmol/pH-kg to 79.3 mmol/pH-kg. It can be inferred that the addition of cations changed the CEC of ash in the WWS by electrostatic adsorption, which can effectively reduce the ABC of WWS’ ash [37].

**Table 1:** The content of ash, surface charge, acid buffering capacity, and cation exchange capacity of the presoaked and raw WWS

Sample	Soaking reagent	Concentration (mmol/L)	Ash (%)	SC^a^ (mv)	CEC^b^ (cmol/kg)	ABC^c^ (mmol/pH-kg)
Raw WWS	No soaking	–	30.5 ± 0.8	− 25.9 ± 1.1	140.2 ± 1.8	226.3 ± 1.2
	H_2_O	–	25.2 ± 1.5	− 24.1 ± 1.3	133.7 ± 2.5	201.4 ± 3.7
	H^+^	30	26.8 ± 1.1	− 22.2 ± 0.3	116.3 ± 1.8	146.7 ± 3.2
	K^+^	30	24.9 ± 1.3	− 23.5 ± 0.5	122.6 ± 2.5	168.9 ± 3.5
	Na^+^	30	27.5 ± 0.7	− 24.7 ± 0.2	118.9 ± 1.4	150.7 ± 2.6
	Ca^2+^	30	23.3 ± 0.5	− 16.3 ± 0.4	135.6 ± 4.6	179.9 ± 2.1
	Mg^2+^	30	24.8 ± 1.2	− 18.5 ± 1.3	109.2 ± 0.9	147.3 ± 4.1
	Zn^2+^	30	26.3 ± 0.4	− 17.6 ± 2.1	96.5 ± 1.3	140.6 ± 1.5
	Fe^2+^	30	25.7 ± 0.2	− 10.5 ± 0.3	80.8 ± 1.1	94.3 ± 2.2
	Fe^2+^	10	23.1 ± 0.1	− 14.7 ± 0.5	97.4 ± 2.8	133.9 ± 1.6
		20	26.3 ± 0.3	− 13.2 ± 0.1	90.5 ± 3.2	119.3 ± 2.3
		30	25.8 ± 0.7	− 11.1 ± 0.0	81.6 ± 1.4	95.6 ± 1.9
		60	24.5 ± 1.1	− 10.8 ± 0.8	74.3 ± 4.2	89.5 ± 2.6
		120	24.3 ± 0.3	− 4.7 ± 0.2	72.4 ± 1.7	79.3 ± 1.0

^a^Surface charge

^b^Cation exchange capacity

^c^Acid buffering capacity

Briefly, the powerful electrostatic force is present on the surface of ash due to the negative charge generated by the isomorphic substitution within layered silicate minerals [36]. Compared with other cations of H^+^, K^+^, Na^+^, Ca^2+^ and Mg^2+^, Fe^2+^ had a greater impact on the negative charges of the ash in WWS due to its specific D orbital alignments and higher valence [38]. According to the selectivity of soil to ions, Fe is also the main inorganic electron acceptor in soil and obviously the best choice to exchange cations on the surface of soil [39]. This electrostatic absorption caused the occurrence of cation exchange reaction on the surface of ash in WWS. Consequently, the introduction of Fe^2+^ caused the ABC of ash in WWS to suffer a severe reduction, and as the amount of Fe^2+^ added increased, the ABC decreased more severely.

### Autohydrolysis of presoaked and raw WWS

#### The pH and composition of the prehydrolysate

During autohydrolysis, the pH of the liquid prehydrolysate generated is a significant parameter. It is related to the solubility of the hemicellulose and indirectly reflects the buffering capacity of ash in WWS [40]. Previous studies have shown that the pH of WWS autohydrolysate after thorough washing of biomass before pretreatment was ~ 4.34 [18]. However, due to the acid buffering capacity of ash in raw WWS, the acidity of prehydrolysate could be reduced, which has lowered the efficiency of autohydrolysis [19]. As shown in Table 2, the pH of prehydrolysate for raw WWS was 5.7. However, the pH of prehydrolysate observably decreased for the WWS that was presoaked with different cations, and the WWS presoaked with Fe^2+^ reached the lowest value (4.0) at the same concentration. It is known that cleavage of hemicellulose acetyl groups is the main drive for lowering pH of the prehydrolysate [20]. Hence, the content of acetyl groups in each sample was determined and shown in the Table 3. It can be seen that the acetyl content of WWS decreased when it was presoaked with Fe^2+^. It is demonstrated that the acid buffering capacity of WWS can be overcome by implementing the cationic presoaking, and successfully forming a weakly acidic medium. The low pH obtained may imply an improvement in the efficiency of autohydrolysis.

**Table 2:** The pH and composition of presoaked and raw WWS prehydrolysate

Sample	Soaking reagent	Concentration (mmol/L)	pH	Carbohydrates (g/L)			Fermentation inhibitors (g/L)			
				Glucose	Xylose	XOS	Formic acid	Acetic acid	Furfural	HMF
Raw WWS	No soaking	–	5.7 ± 0.1	0.0 ± 0.0	0.7 ± 0.1	2.9 ± 0.1	0.2 ± 0.0	0.7 ± 0.1	0.0 ± 0.0	0.0 ± 0.0
	H_2_O	–	5.0 ± 0.3	0.0 ± 0.0	0.3 ± 0.2	3.0 ± 0.1	0.2 ± 0.0	0.7 ± 0.1	0.0 ± 0.0	0.0 ± 0.0
	H^+^	30	4.9 ± 0.1	0.1 ± 0.1	0.3 ± 0.3	3.3 ± 0.3	0.2 ± 0.1	0.7 ± 0.0	0.1 ± 0.1	0.0 ± 0.1
	K^+^	30	5.1 ± 0.0	0.0 ± 0.0	0.2 ± 0.1	3.2 ± 0.2	0.1 ± 0.0	0.7 ± 0.0	0.0 ± 0.0	0.0 ± 0.0
	Na^+^	30	4.8 ± 0.0	0.1 ± 0.0	0.4 ± 0.0	2.9 ± 0.3	0.0 ± 0.0	0.7 ± 0.1	0.0 ± 0.0	0.0 ± 0.0
	Ca^2+^	30	4.6 ± 0.1	0.2 ± 0.0	0.5 ± 0.2	3.1 ± 0.2	0.2 ± 0.1	0.8 ± 0.0	0.3 ± 0.1	0.0 ± 0.0
	Mg^2+^	30	4.4 ± 0.0	0.2 ± 0.1	0.7 ± 0.0	3.1 ± 0.1	0.3 ± 0.2	0.8 ± 0.1	0.3 ± 0.2	0.0 ± 0.0
	Zn^2+^	30	4.1 ± 0.0	0.3 ± 0.0	1.3 ± 0.0	4.0 ± 0.1	0.5 ± 0.0	1.1 ± 0.0	1.1 ± 0.1	0.1 ± 0.0
	Fe^2+^	30	4.0 ± 0.0	0.3 ± 0.2	0.9 ± 0.1	4.9 ± 0.3	0.9 ± 0.0	1.5 ± 0.0	1.1 ± 0.1	0.1 ± 0.0
	Fe^2+^	10	4.0 ± 0.0	0.1 ± 0.1	1.1 ± 0.3	4.3 ± 0.4	0.8 ± 0.1	1.3 ± 0.1	1.0 ± 0.0	0.1 ± 0.0
		20	4.0 ± 0.2	0.1 ± 0.0	1.0 ± 0.1	4.6 ± 0.1	0.9 ± 0.0	1.3 ± 0.2	1.0 ± 0.1	0.2 ± 0.1
		30	4.0 ± 0.1	0.3 ± 0.1	0.8 ± 0.0	5.1 ± 0.3	0.9 ± 0.0	1.5 ± 0.0	1.2 ± 0.0	0.2 ± 0.0
		60	3.9 ± 0.0	0.8 ± 0.2	0.7 ± 0.1	5.6 ± 0.2	0.9 ± 0.1	1.6 ± 0.0	1.2 ± 0.0	0.2 ± 0.0
		120	3.8 ± 0.1	1.0 ± 0.0	0.7 ± 0.2	6.2 ± 0.2	1.1 ± 0.1	1.8 ± 0.0	1.3 ± 0.1	0.3 ± 0.1

**Table 3:** Effects of presoaked WWS and raw WWS on autohydrolysis

Sample	Soaking reagent	Concentration (mmol/L)	Composition (%)				Recovery (%)		Removal (%)	
			Glucan	Xylan	Lignin	Acetyl	Solid	Glucan	Xylan	Lignin
Raw WWS	No soaking	–	43.7 ± 0.2	8.4 ± 0.6	24.5 ± 0.1	0.7 ± 0.1	60.9 ± 0.4	94.3 ± 0.5	61.7 ± 0.2	16.5 ± 0.2
	H_2_O	–	48.9 ± 0.3	7.6 ± 0.1	24.4 ± 0.2	0.4 ± 0.0	59.4 ± 0.3	96.7 ± 1.7	66.8 ± 0.2	19.8 ± 0.3
	H^+^	30	48.2 ± 0.5	7.6 ± 0.2	24.2 ± 0.4	0.3 ± 0.1	56.1 ± 0.6	95.8 ± 1.4	69.3 ± 0.6	21.6 ± 0.3
	K^+^	30	46.7 ± 0.3	7.3 ± 0.5	24.0 ± 0.2	0.4 ± 0.1	57.2 ± 1.1	94.7 ± 0.6	68.5 ± 0.5	20.1 ± 0.5
	Na^+^	30	49.1 ± 0.4	8.9 ± 0.2	24.3 ± 0.6	0.4 ± 0.2	56.8 ± 0.5	98.7 ± 1.1	62.2 ± 0.3	22.7 ± 0.2
	Ca^2+^	30	48.1 ± 0.2	7.8 ± 0.6	22.5 ± 0.1	0.4 ± 0.0	57.4 ± 0.8	97.7 ± 1.4	66.6 ± 0.3	21.6 ± 0.1
	Mg^2+^	30	46.5 ± 0.3	7.1 ± 0.7	25.5 ± 0.2	0.3 ± 0.1	58.3 ± 0.3	96.2 ± 0.7	69.1 ± 0.1	23.4 ± 0.4
	Zn^2+^	30	50.5 ± 0.3	5.4 ± 0.5	26.5 ± 0.1	0.2 ± 0.1	50.3 ± 0.9	90.1 ± 1.3	79.7 ± 0.2	25.3 ± 0.6
	Fe^2+^	30	53.4 ± 0.6	4.8 ± 0.3	27.1 ± 0.3	0.2 ± 0.0	49.4 ± 0.4	94.2 ± 0.4	81.9 ± 0.4	27.6 ± 0.3
	Fe^2+^	10	54.7 ± 0.7	4.3 ± 0.1	25.4 ± 0.2	0.3 ± 0.0	49.4 ± 0.6	93.2 ± 0.8	80.2 ± 0.5	24.9 ± 0.1
		20	53.7 ± 0.2	4.4 ± 0.5	26.5 ± 0.3	0.2 ± 0.1	50.9 ± 0.4	96.8 ± 1.1	81.5 ± 0.2	26.3 ± 0.4
		30	53.6 ± 0.1	4.9 ± 0.5	27.2 ± 0.1	0.2 ± 0.1	49.7 ± 0.7	94.9 ± 0.9	82.7 ± 0.4	27.4 ± 0.5
		60	54.1 ± 0.5	4.7 ± 0.2	26.4 ± 0.6	0.1 ± 0.2	47.4 ± 0.4	93.9 ± 1.5	83.1 ± 0.8	29.8 ± 0.3
		120	54.0 ± 0.4	4.6 ± 0.4	27.1 ± 0.4	0.0 ± 0.0	45.9 ± 0.3	87.8 ± 2.2	84.7 ± 0.7	30.2 ± 0.3

The concentration of each prehydrolysate was measured and is shown in Table 2. It was found that the concentration of xylose and xylooligosaccharides in the prehydrolysate of the raw WWS was 0.7 g/L and 2.9 g/L, respectively. When the WWS was presoaked with the same concentration of H^+^, K^+^, Na^+^, Ca^2+^, Mg^2+^ Zn^2+^, Fe^2+^, the concentration of the xylose was 0.3 g/L, 0.2 g/L, 0.4 g/L, 0.5 g/L, 0.7 g/L, 1.3 g/L, 0.9 g/L and the concentration of the XOS was 3.3 g/L, 3.2 g/L, 2.9 g/L, 3.1 g/L, 3.1 g/L, 4.0 g/L, 4.9 g/L, respectively. Both xylose and XOS were derived from the depolymerization of xylan in hemicellulose during autohydrolysis [11, 41]. The presoaking with different cations has reduced the acid buffering capacity of WWS, and thus lowered the pH of the prehydrolysate, which may be the key factor for the production of more xylose and XOS. In addition, the concentration of the fermentation inhibitors was also higher than that in raw WWS prehydrolysate. For instance, when the WWS was presoaked with same concentration of Zn^2+^ and Fe^2+^, the formation of inhibitors was increased compared to that of WWS prehydrolysate without soaking. This may be due to lower acidity of the prehydrolysate in autohydrolysis facilitating the depolymerization reaction of carbohydrates [19]. It has also been reported that Zn^2+^ and Fe^2+^ possessed unique characteristics in catalytic transformations, which can selectively catalyze the depolymerization of carbohydrates into oligosaccharide or dehydration of monosaccharide into fermentation inhibitors [42, 43]. It can be inferred that the buffering capacity of the ash from WWS was decreased, which improved the acidity of the autohydrolysate, allowing for xylan to depolymerize into xylose or XOS during autohydrolysis. However, the increasing acid mediums can force the xylose to further cyclodehydrate into fermentation inhibitors at high temperature (180 °C), such as furfural and formic acid.

#### Chemical composition, glucan recovery, xylan and lignin removal from autohydrolyzed WWS

The chemical composition of the autohydrolyzed WWS was determined by NREL standard method. As shown in Table 3, xylan removal of WWS without presoaking was only 61.7%, this value was decidedly lower than that of presoaked WWS. The xylan removal was 66.8%, 69.3%, 68.5%, 62.2%, 66.6%, 69.1% and 79.7% after the WWS was presoaked with H_2_O, 30 mM of H^+^, K^+^, Na^+^ Ca^2+^, Mg^2+^ and Zn^2+^ solutions, respectively. When the WWS was presoaked with increased concentration of Fe^2+^ from 10 to 120 mM, the xylan removal was raised from 71.6 to 83.7%. Compared with other cations, Fe^2+^ has a better effect on xylan removal for WWS. The reason for this results may be the acidity of prehydrolysate was improved by breaking the acid buffering capacity of ash, which was attributed to the addition of Fe^2+^. The improved acidity of the acid medium resulted more hydronium ions in the prehydrolysate for the better xylan removal [19].

Table 3 also displayed that the removal of lignin increased from 16.5% of raw WWS after presoaking. Specifically, delignification of 120 mM Fe^2+^ presoaked WWS was nearly twofold higher than that of the raw WWS (30.2%). During autohydrolysis, lignin undergoes an unknown extent of simultaneous depolymerization and repolymerization reactions upon heating [11, 41]. Addition of Fe^2+^ from 10 to 120 mM caused lignin removal to increase from 24.9 to 30.2%. This is likely due to iron’s ability to negate the acid buffering of the ash in the pretreatment medium. The acidity enhanced by eliminating the buffering effect could be facilitating additional decomposition of β-*O*-4 bonds in lignin, enhancing the degree of delignification [44, 45]. Moreover, Fe^2+^ exchanged on ash surface is easily complexed with depolymerized lignin during autohydrolysis, which has also facilitated delignification [19]. It could provide a morphological benefit to further enzymatic hydrolysis in the form of collapse of fiber pores by delignification.

### Enzymatic hydrolysis of autohydrolyzed WWS

**Fig. 2:**
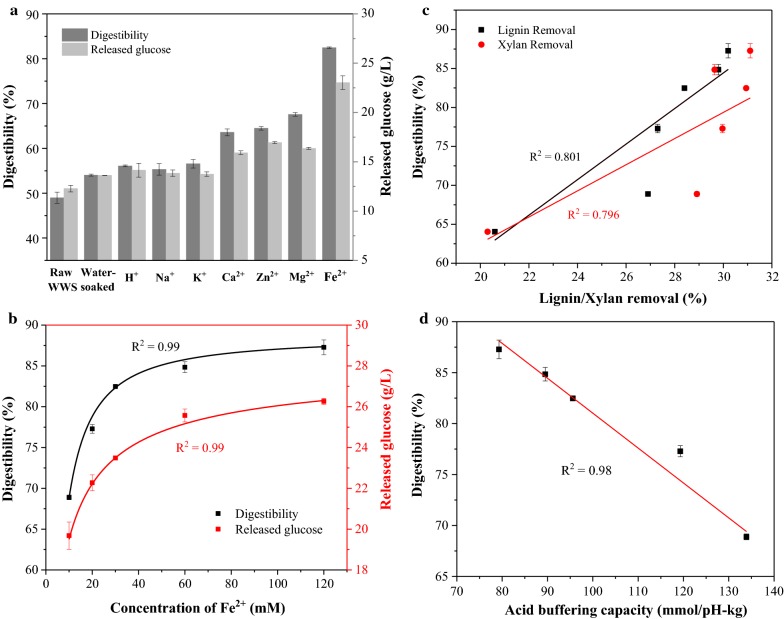
Effect of different cations (**a**) and different concentrations of Fe^2+^ (**b**) soaking on the digestibility and released glucose of the autohydrolyzed WWS in enzymatic hydrolysis, and the respective relationship between xylan/lignin (**c**) and acid buffering capacity (**d**) with digestibility of autohydrolyzed WWS after different concentrations of Fe^2+^ presoaking

The enzymatic hydrolysis of autohydrolyzed WWS was carried out to evaluate the effect of presoaking with different cations on the autohydrolysis of WWS, the glucan conversion was represented by enzymatic digestibility. Figure 2a displays the enzymatic digestibility and glucose release of raw WWS and that which was presoaked with different cations. The enzymatic digestibility of autohydrolyzed WWS without presoaking was just 48.9%. However, this value increased to different extents by presoaking, specifically increased to 54.04% (H_2_O), 56.1% (H^+^), 55.3% (Na^+^), 56.5% (K^+^), 63.6% (Ca^2+^), 64.5% (Mg^2+^) and 82.5% (Fe^2+^) after 72 h of enzymatic hydrolysis. When the concentration of Fe^2+^ increased to 120 mM, the enzymatic digestibility reached 86.3%, which can be seen in Fig. 2b. These results demonstrated that enzymatic digestibility of autohydrolyzed WWS can indeed be improved by implementing presoaking with cations. It has been reported that lignin and hemicellulose are major obstacles to enzymatic hydrolysis, therefore improving their removal and general structural disruption logically contributed to enhancing enzymatic kinetics [46, 47]. For example, the WWS after presoaking with 120 mM Fe^2+^ had xylan removal of 83.7% and lignin removal of 30.2% during the autohydrolysis, while the original WWS was only 68.1% and 16.5%. As shown in Fig. 2c, the removal of xylan and lignin is positively correlated with the enzymatic digestibility. Presoaking appeared to have induced a change in ABC of ash in WWS, and the removal of xylan and lignin was thus improved. As shown in Fig. 2d, the decrease in ABC was indirectly contributed to improving the enzymatic digestibility, which presented a negative correlation (*R*^2^ = 0.98) between the ABC of presoaked WWS and enzymatic digestibility.

### Structural property changes around presoaking and autohydrolysis of WWS

#### Cellulase accessibility

**Fig. 3:**
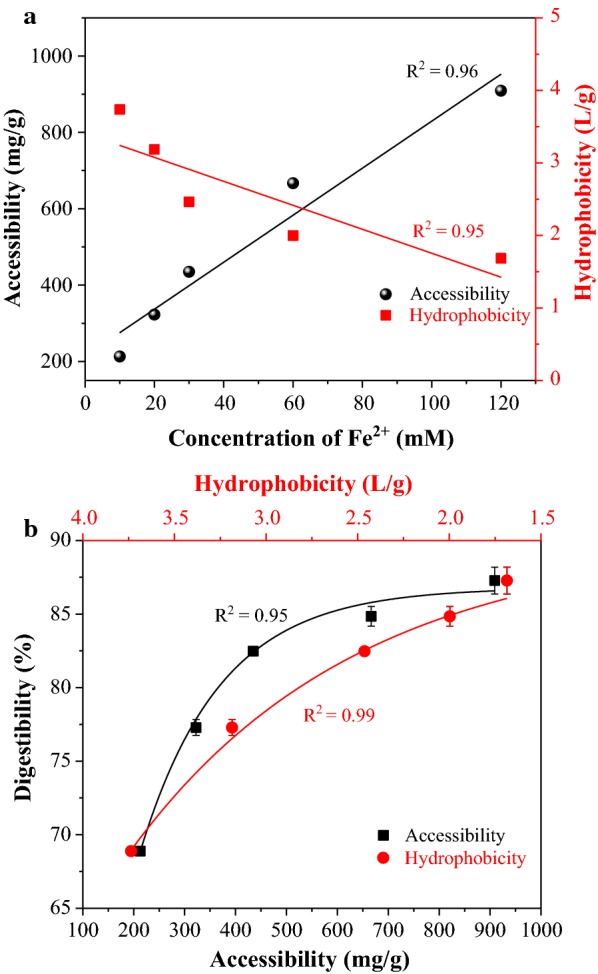
Effect of different concentrations of Fe^2+^ presoaking on the accessibility or hydrophobicity of autohydrolyzed WWS (**a**), and the respective relationship between accessibility or hydrophobicity with digestibility of autohydrolyzed WWS during enzymatic hydrolysis (**b**)

The accessibility of cellulose to cellulase has always been one of the major limitations to enzymatic hydrolysis [48]. To investigate why presoaking with Fe^2+^ increased the enzymatic hydrolysis of autohydrolyzed WWS, the Direct Rad 28 dye adsorption method was used to estimate the accessibility of cellulase to cellulose in the presoaked WWS. The results of the adsorption assay revealed that the accessibility of cellulose significantly increased after WWS was presoaked with Fe^2+^, which can be seen in Fig. 3a. For example, the accessibility of the presoaked WWS increased from 212.7 mg/g (mg dry/g substrate) to 909.1 mg/g when the concentration of Fe^2+^ increased from 10 to 120 mM. For comparison, accessibility of raw WWS pretreatment residue was only 144.9 mg/g. This observation suggests that Fe^2+^ enhances the efficiency of pretreatment by decreasing the ABC of WWS, which enhanced the lignin/xylan removal and directly affected the tight structure of the WWS cell walls thus enhancing the accessibility of cellulose [32]. It was also found that the correlation between enzymatic digestibility and the accessibilities of the autohydrolyzed WWS was linear (*R*^2^ = 0.99), explaining that presoaking contributed to increasing accessibility.

#### Hydrophobicity

A previous discussion about the hydrophobicity of lignin in autohydrolyzed residues inhibiting enzymes through non-productive binding has been reported [49]. One method for evaluating substrate hydrophobicity is the Rose Bengal partitioning method [50]. In this work, the hydrophobicity of all autohydrolyzed WWS was thus determined using Rose Bengal dye to evaluate the relationship between hydrophobicity and enzymatic digestibility. As shown in Fig. 3a, when Fe^2+^ concentration increased from 10 to 120 mM, the hydrophobicity of the autohydrolyzed WWS decreased from 3.7 to 1.7 L/g. It has been reported that lignin was a hydrophobic polymer that can non-specifically adsorb cellulases, resulting in less free enzymes for cellulose degrading during enzymatic hydrolysis [51]. The depolymerization of lignin in WWS autohydrolysis was intensified due to the increase in Fe^2+^ concentration of presoaking, which decreased the surface hydrophobicity of WWS. Therefore, it was helpful towards enhancing the binding tendency between cellulase and autohydrolyzed residues and promoting enzymatic hydrolysis efficiency. As shown in Fig. 3b, the hydrophobicity of the autohydrolyzed WWS was relatively negative correlated (*R*^2^ = 0.99) with enzymatic digestibility. The highest enzymatic hydrolysis efficiency achieved is from the material with the lowest measured surface hydrophobicity. This result also demonstrated that hydrophobicity is a critical factor towards enzymatic hydrolysis.

## Conclusions

The ash in WWS was found to be detrimental to autohydrolysis because of its acid buffering capacity. Presoaking with cations was shown to eliminate this negative effect for WWS prior to autohydrolysis, and Fe^2+^ is the most effective cation due to its particular valence/orbit to exchange with the other cations on the surface of the ash, which caused the reduction in acid buffering capacity. The results show that the WWS can be made digestible by cellulases if effort is made to eliminate the ABC of ash in raw WWS, opening the door for this material to be converted into a valuable biochemical.

## Data Availability

All data generated and analyzed in this study are included in this published article.

## Abbreviations

WWS: Waste wheat straw
SC: Surface charge
CEC: Cations exchange capacity
ABC: Acid buffering capacity
NREL: National Renewable Energy Laboratory
XOS: Xylo-oligosaccharides
HPLC: High-performance liquid chromatography
HMF: Hydroxymethylfurfural

## References

[1] Lynd LR, Cushman JH, Nichols RJ, Wyman CE (1991). Fuel ethanol from cellulosic biomass. Science.

[2] Lynd LR, Laser MS, Bransby D, Dale BE, Davison B, Hamilton R, Himmel M, Keller M, McMillan JD, Sheehan J, Wyman CE (2008). How biotech can transform biofuels. Nat Biotechnol.

[3] Satari B, Karimi K, Kumar R (2019). Cellulose solvent-based pretreatment for enhanced second-generation biofuel production: a review. Sustain Energy Fuels.

[4] Xie H, Zhang D, Mao G, Wang F, Song A (2018). Availability of lignocellulose from forestry waste for use as a biofuel in China. 3 Biotech.

[5] Lynd LR, Wyman CE, Gerngross TU (1999). Biocommodity engineering. Biotechnol Prog.

[6] Himmel ME, Ding SY, Johnson DK, Adney WS, Nimlos MR, Brady JW, Foust TD (2007). Biomass recalcitrance: engineering plants and enzymes for biofuels production. Science.

[7] Cao T, Jiang B, Gu F, Wu W, Jin Y (2018). Effects of green liquor (GL) and sodium carbonate (SC) pretreatment on structural characteristics of wheat stem lignin. J Wood Chem Technol.

[8] Mosier N, Wyman C, Dale B, Elander R, Lee YY, Holtzapple M, Ladisch M (2005). Features of promising technologies for pretreatment of lignocellulosic biomass. Bioresour Technol.

[9] Wyman CE, Dale BE, Balan V, Elander RT, Holtzapple MT, Ramirez RS, Ladisch MR, Mosier NS, Lee YY, Gupta R, Thomas SR, Hames BR, Warner R, Kumar R (2013). Comparative performance of leading pretreatment technologies for biological conversion of corn stover, poplar wood, and switchgrass to sugars.

[10] Gupta VK, Tuohy MG (2013). Progress in physical and chemical pretreatment of lignocellulosic biomass. Biofuel technologies.

[11] Zhuang X, Wang W, Yu Q, Qi W, Wang Q, Tan X, Zhou G, Yuan Z (2016). Liquid hot water pretreatment of lignocellulosic biomass for bioethanol production accompanying with high valuable products. Bioresour Technol.

[12] Wang W, Zhuang X, Yuan Z, Yu Q, Qi W, Wang Q, Tan X (2012). High consistency enzymatic saccharification of sweet sorghum bagasse pretreated with liquid hot water. Bioresour Technol.

[13] Lu H, Liu S, Zhang M, Meng F, Shi X, Yan L (2016). Investigation of the strengthening process for liquid hot water pretreatments. Energy Fuel.

[14] Wu W, Jiang B, Yang L, Jin Y (2018). Isolation of lignin from Masson’s pine by liquid-liquid extraction based on complete dissolution in NaOH aqueous solution. BioResources.

[15] Hendriks AT (2009). Pretreatments to enhance the digestibility of lignocellulosic biomass. Bioresour Technol.

[16] Zhang H, Xu S, Wu S (2013). Enhancement of enzymatic saccharification of sugarcane bagasse by liquid hot water pretreatment. Bioresour Technol.

[17] He Y, Fang Z, Zhang J, Li X, Bao J (2014). De-ashing treatment of corn stover improves the efficiencies of enzymatic hydrolysis and consequent ethanol fermentation. Bioresour Technol.

[18] Huang C, Wu X, Huang Y, Lai C, Li X, Yong Q (2016). Prewashing enhances the liquid hot water pretreatment efficiency of waste wheat straw with high free ash content. Bioresour Technol.

[19] Wu X, Huang C, Tang W, Huang C, Lai C, Yong Q (2018). Use of metal chlorides during waste wheat straw autohydrolysis to overcome the self-buffering effect. Bioresour Technol.

[20] Han Q, Jin Y, Jameel H, Chang H, Phillips R, Park S (2015). Autohydrolysis pretreatment of waste wheat straw for cellulosic ethanol production in a co-located straw pulp mill. Appl Biochem Biotech..

[21] Six J, Bossuyt H, Degryze S, Denef K (2004). A history of research on the link between (micro)aggregates, soil biota, and soil organic matter dynamics. Soil Tillage Res.

[22] Lloyd TA, Wyman CE (2004). Predicted effects of mineral neutralization and bisulfate formation on hydrogen ion concentration for dilute sulfuric acid pretreatment. Appl Biochem Biotechnol.

[23] Cai J, Luo W, Liu H, Feng X, Zhang Y, Wang R, Xu Z, Zhang Y, Jiang Y (2017). Precipitation-mediated responses of soil acid buffering capacity to long-term nitrogen addition in a semi-arid grassland. Atmos Environ.

[24] Liu C, Wyman CE (2006). The enhancement of xylose monomer and xylotriose degradation by inorganic salts in aqueous solutions at 180 degrees C. Carbohydr Res.

[25] Monavari S, Galbe M, Zacchi G (2011). The influence of ferrous sulfate utilization on the sugar yields from dilute-acid pretreatment of softwood for bioethanol production. Bioresour Technol.

[26] Sorensen A, Teller PJ, Hilstrom T, Ahring BK (2008). Hydrolysis of Miscanthus for bioethanol production using dilute acid presoaking combined with wet explosion pre-treatment and enzymatic treatment. Bioresour Technol.

[27] He J, Huang C, Lai C, Huang C, Yong Q (2017). Relations between moso bamboo surface properties pretreated by kraft cooking and dilute acid with enzymatic digestibility. Appl Biochem Biotechnol.

[28] Hendershot WH, Duquette M (1986). A simple barium chloride method for determining cation exchange capacity and exchangeable cations. Soil Sci Soc Am J.

[29] Nelson PN, Su N (2010). Soil pH buffering capacity: a descriptive function and its application to some acidic tropical soils. Aust J Soil Res.

[30] Wu X, Huang C, Zhai S, Liang C, Huang C, Lai C, Yong Q (2018). Improving enzymatic hydrolysis efficiency of wheat straw through sequential autohydrolysis and alkaline post-extraction. Bioresour Technol.

[31] Sluiter JB, Ruiz RO, Scarlata CJ, Sluiter AD, Templeton DW (2010). Compositional analysis of lignocellulosic feedstocks. 1. Review and description of methods. J Agric Food Chem.

[32] Wiman M, Dienes D, Hansen MAT, van der Meulen T, Zacchi G, Lidén G (2012). Cellulose accessibility determines the rate of enzymatic hydrolysis of steam-pretreated spruce. Bioresour Technol.

[33] Gessner A, Waicz R, Lieske A, Paulke B-R, Mader K, Muller RH (2000). Nanoparticles with decreasing surface hydrophobicities: influence on plasma protein adsorption. Int J Pharm.

[34] Oorts K, Vanlauwe B, Pleysier J, Merckx R (2004). A new method for the simultaneous measurement of pH-dependent cation exchange capacity and pH buffering capacity. Soil Sci Soc Am J.

[35] Olphen HV (1964). An introduction to clay colloid chemistry. Science.

[36] Kraepiel AML, Keller K, Morel FMM (1999). A model for metal adsorption on montmorillonite. J Colloid Interface Sci.

[37] Breemen NV, Burrough PA, Velthorst EJ, Van Dobbent HF, de Witt T, Ridder TB, Reijnders HFR (1982). Soil acidification from atmospheric ammonium sulphate in forest canopy throughfall. Nature.

[38] Ma C, Eggleton RA (1999). Cation exchange capacity of kaolinite. Clay Clay Miner.

[39] Hou X, Li Y, Pan Y, Jin Y, Xiao H (2018). Controlled release of agrochemicals and heavy metal ion capture dual-functional redox-responsive hydrogel for soil remediation. Chem Commun.

[40] Guzman MV, Garcia JMP, Santos GA, Maroto JMR, Alonso CV, Lahoz CG (2015). Effects of the buffering capacity of the soil on the mobilization of heavy metals. Equilibrium and kinetics. Chemosphere.

[41] Ko JK, Kim Y, Ximenes E, Ladisch MR (2015). Effect of liquid hot water pretreatment severity on properties of hardwood lignin and enzymatic hydrolysis of cellulose. Biotechnol Bioeng.

[42] Liu L, Sun J, Cai C, Wang S, Pei H, Zhang J (2009). Corn stover pretreatment by inorganic salts and its effects on hemicellulose and cellulose degradation. Bioresour Technol.

[43] Wang H, Zhang L, Deng T, Ruan H, Hou X, Cort JR, Yang B (2016). ZnCl2 induced catalytic conversion of softwood lignin to aromatics and hydrocarbons. Green Chem.

[44] Huang C, Lin W, Lai C, Li X, Jin Y, Yong Q (2019). Coupling the post-extraction process to remove residual lignin and alter the recalcitrant structures for improving the enzymatic digestibility of acid-pretreated bamboo residues. Bioresour Technol.

[45] Huang C, Su Y, Shi J, Yuan C, Zhai S, Yong Q (2019). Revealing the effects of centuries of ageing on the chemical structural features of lignin in archaeological fir woods. New J Chem.

[46] Tao P, Zhang Y, Wu Z, Liao X, Nie S (2019). Enzymatic pretreatment for cellulose nanofibrils isolation from bagasse pulp: transition of cellulose crystal structure. Carbohydr Polym..

[47] Yang B, Wyman CE (2004). Effect of xylan and lignin removal by batch and flowthrough pretreatment on the enzymatic digestibility of corn stover cellulose. Biotechnol Bioeng.

[48] Yu H, You Y, Lei F, Liu Z, Zhang W, Jiang J (2015). Comparative study of alkaline hydrogen peroxide and organosolv pretreatments of sugarcane bagasse to improve the overall sugar yield. Bioresour Technol.

[49] Huang C, Ma J, Liang C, Li X, Yong Q (2018). Influence of sulfur dioxide-ethanol-water pretreatment on the physicochemical properties and enzymatic digestibility of bamboo residues. Bioresour Technol.

[50] Lukowski G, Miiller RH, Miiller BW, Dittgen M (1992). Acrylic acid copolymer nanoparticles for drug delivery: I. Characterization of the surface properties relevant for in vivo organ distribution. Int J Pharm.

[51] Gu L, Jiang B, Song J, Jin Y, Xiao H (2018). Effect of lignin on performance of lignocellulose nanofibrils for durable superhydrophobic surface. Cellulose.

